# Current and next-generation formulation strategies for inactivated polio vaccines to lower costs, increase coverage, and facilitate polio eradication

**DOI:** 10.1080/21645515.2022.2154100

**Published:** 2022-12-28

**Authors:** Prashant Kumar, Christopher Bird, David Holland, Sangeeta B. Joshi, David B. Volkin

**Affiliations:** Department of Pharmaceutical Chemistry, Vaccine Analytics and Formulation Center, University of Kansas, Lawrence, KS, USA

**Keywords:** Inactivated polio vaccine, combination vaccines, formulation, stability, D-antigen ELISA, dose-sparing, adjuvants, vaccine delivery

## Abstract

Implementation of inactivated polio vaccines (IPV) containing Sabin strains (sIPV) will further enable global polio eradication efforts by improving vaccine safety during use and containment during manufacturing. Moreover, sIPV-containing vaccines will lower costs and expand production capacity to facilitate more widespread use in low- and middle-income countries (LMICs). This review focuses on the role of vaccine formulation in these efforts including traditional Salk IPV vaccines and new sIPV-containing dosage forms. The physicochemical properties and stability profiles of poliovirus antigens are described. Formulation approaches to lower costs include developing multidose and combination vaccine formats as well as improving storage stability. Formulation strategies for dose-sparing and enhanced mucosal immunity include employing adjuvants (e.g. aluminum-salt and newer adjuvants) and/or novel delivery systems (e.g. ID administration with microneedle patches). The potential for applying these low-cost formulation development strategies to other vaccines to further improve vaccine access and coverage in LMICs is also discussed.

## Introduction to poliovirus and polio vaccines

Poliomyelitis is a disabling and life-threatening disease caused by the highly infectious poliovirus.^1^ Poliovirus infections primarily occur in children below 5 years of age and are infamous for causing acute flaccid paralysis. Person-to-person transmission via the fecal-oral route is well-established with the virus infecting the intestinal tissue. Although most cases of poliomyelitis are asymptomatic or mildly symptomatic, showing signs of only a general viral illness,^2^ the virus can move from the intestines into the blood stream, exposing other tissues to infection. Cells in the nervous system are susceptible to poliovirus infection due to their expression of the CD155 receptor in which the membrane-distal V-type domain binds to poliovirus. In the most severe cases, the disease can fatally interfere with breathing or swallowing.^2^ The earliest historical accounts of poliomyelitis date back to 1400 BCE in Ancient Egypt, and into the 20^th^ century it was recognized as a major public health threat in the United States.^3^ The danger of permanently paralyzing children and adults made it among the most feared infectious diseases, notably confining a future US president Franklin Roosevelt to a wheelchair.^4^

**Figure 1.**
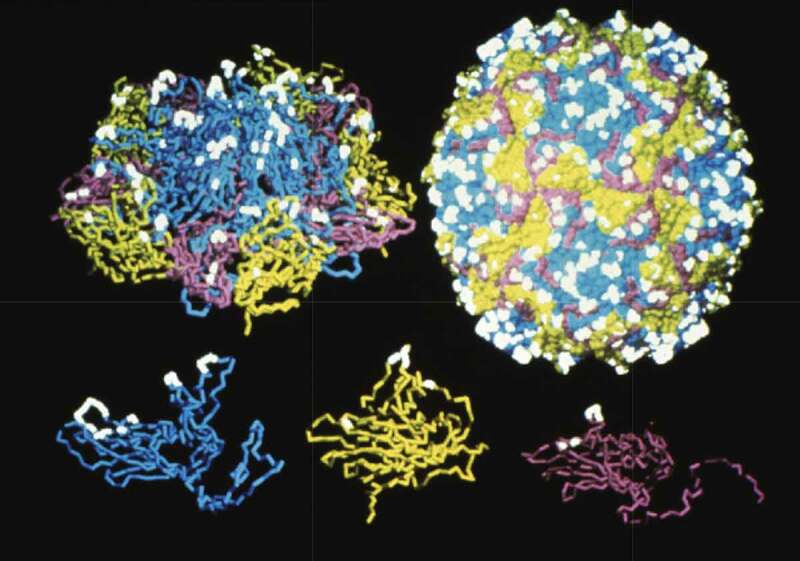
Schematic of the structure of poliovirus capsid proteins, the protomer subunit and the assembled poliovirus. The D-antigen epitope sites that induce protective immunity are highlighted (white). The viral proteins displayed in the bottom panel are VP1 (Blue), VP2 (Yellow), and VP3 (Purple) which combine with the internal VP4 protein (not shown) to create the protomer subunit (top left panel). The assembled poliovirus (top right panel) is a 30 nm icosahedron-shaped particle consisting of 60 subunits and the RNA genome. Figure reproduced from Vaccines 2018, Chapter 48^17^ with permission from Elsevier.

Poliovirus is an enterovirus and classified as a member of the picornavirus family. The poliovirus capsid is non-enveloped and consists of viral proteins surrounding a genome of a single stranded messenger sense RNA. As shown in Figure 1, the icosahedral viral capsid is 30 nm in size and consists of 60 protomer subunits made of 4 viral proteins: VP1, VP2, VP3, and VP4. In addition, there are also three strains of poliovirus termed PV1, PV2, and PV3.^5^ In the late 1950s, scientists demonstrated that each serotype of poliovirus can contain C- or D-antigenic sites. The D-antigen is associated with native infectious particles that induce generation of protective antibodies, while the C-antigen content correlates with noninfectious particles.^6^ The D-antigen can convert to the C-antigen by exposure to a variety of environmental stress conditions including high temperatures (e.g., 60°C), ultraviolet light, drying, mercury-containing compounds, phenol and high solution pH.^7,8^

Two types of polio vaccines were developed in the 1950s and are still currently widely used worldwide including (1) an orally administered vaccine containing live, attenuated poliovirus antigens (referred to as OPV or Sabin), and (2) a parenterally administered vaccine containing inactivated poliovirus antigens (referred to as IPV or Salk). Sabin OPV and Salk IPV vaccines have led to dramatic public health improvements, for example, yearly wild type polio cases have decreased from hundreds of thousands in the 1940s and 50s, to just 21 cases in 2018. These highly efficacious vaccines have led to the ambitious goal of targeting polio disease for worldwide eradication. PV1 is the only wild-type poliovirus that has yet to be eradicated. The final cases of wild-type PV2 and PV3 were observed in 1999 and 2012, respectively, and were certified as eradicated in 2015 and 2019, respectively.^9^ Currently, the only remaining countries impacted by wild-type polio are Pakistan and Afghanistan, although political instability and the COVID-19 pandemic have unfortunately hampered vaccination efforts, leading to a rise in cases each year from 2016 to 2021.^10,11^ In addition, isolated cases still occur from reversion of the attenuated Sabin vaccine strains (see below), including, for example, a recent 2022 report of polio infection in an unvaccinated young adult in Rockland County, New York, USA.^12^ Despite the enormous success of polio vaccination efforts in improving public health, there are inherent limitations to both the current Sabin OPV and Salk IPV polio vaccines in terms of accomplishing complete worldwide eradication of polio as discussed below.

### Sabin OPV vaccines

An orally administered, live polio vaccine was developed by a team led by Albert Sabin in 1957, and it became the most widely distributed polio vaccine in the 20^th^ century. The first example of a live, orally administered polio vaccine was developed by a team led by Hilary Koprowski in 1950, and this attenuated poliovirus strain was the starting point for the subsequent development of attenuated Sabin strains. The oral polio vaccine (OPV) contains live, attenuated poliovirus strains of each of three serotypes (Types 1, 2, 3). Attenuation of polioviruses was achieved by adapting the virus to grow in a series of non-human cells, causing key mutations resulting in loss of virulence in humans, i.e., selecting for strains that were capable of infecting intestinal tissue but incapable of infecting nervous tissue such as the brain and spinal cord.^13^ The ability of OPV to replicate in the body improves protective immune responses that can induce lifelong immunity and confer incidental contact immunity through accidental environmental exposure to OPV shed in the stool. Furthermore, the improved mucosal immunity in the intestines provided by OPV contributes to improved community protection by preventing the spread of wild-type viruses to unvaccinated individuals.^13^ Finally, the orally delivered OPV vaccine is easier and less costly to administer compared to parenteral injection of IPV vaccines (see below), especially since it can be carried out by volunteers in addition to trained medical professionals.

A major drawback of Sabin OPV vaccine, however, is the rare ability of the attenuated vaccine virus to mutate and regain neurovirulence while propagating in the intestines, leading to vaccine-associated paralytic poliomyelitis (VAPP). VAPP is estimated to occur at one per 4.7 per million births,^13^ affects vaccine recipients and their close contacts, but is not associated with larger-scale outbreaks. More rarely, the attenuated vaccine virus can mutate in humans to regain both neurovirulence and sustained transmissibility, referred to as circulating vaccine-derived poliovirus (cVDPV), an event capable of leading to larger-scale polio outbreaks. Another drawback to OPV vaccinations is that the seroconversion rates for OPV1 and OPV3 are lower when in combination with OPV2 than they are for monovalent vaccines or a bivalent vaccine for types 1 and 3.^14^ Upon eradication of the wild type 2 polio virus in 2015, the World Health Organization (WHO) no longer recommends trivalent OPV for routine immunization. Simply removing type 2 OPV from oral vaccination schedules, however, would create a gap in type 2 population immunity thereby creating a population that is susceptible to type 2 cVDPV or the remote possibility of contracting wild poliovirus type 2. For these reasons, the WHO recommends at least one dose of IPV in childhood immunization schedule. Type 2 cVDPV has recently become a growing problem, with global cases increasing from 71 (in 2018) to 366 (in 2019) and 1037 (in 2020). In the event of a cVDPV outbreak, OPV is used to prevent spread within the community. In late 2020, the WHO approved the emergency use of a novel type 2 OPV (nOPV2) which provides equivalent protection but is less likely to revert to virulence.^15^ The nOPV2 is antigenically indistinguishable from OPV2 but is more genetically stable and was prepared by codon-deoptimization to further attenuate OPV2.^16^

### Salk IPV vaccines

An intramuscularly (IM) injected, inactivated polio vaccine was developed by a team led by Jonas Salk in 1955. The production of inactivated polio vaccine (IPV) employs a formalin treatment step to inactivate three strains of wildtype polio virus: Mahoney (IPV1), MEFI (IPV2), and Saukett (IPV3).^17^ The production of formalin-inactivated polio viruses therefore involves growing large quantities of the virulent virus (see next section), necessitating extensive and meticulously managed safety and biocontainment protocols. When properly manufactured, IPV is safe and efficacious with mild side effects. Upon initial approval of IPV vaccines for use in the USA in 1955, a devastating event occurred at one of the vaccine manufacturers known as the Cutter Incident, in which some of the produced IPV vaccine was not properly inactivated. This resulted in the administration of a live wild-type poliovirus causing 220,000 infections, 10 deaths and leaving 164 paralyzed after vaccination.^18,19^ The trauma of the Cutter incident led to the implementation of modern FDA regulations for vaccine manufacturing thereby ensuring safe IPV vaccine production.^19^ In terms of immune responses, although IM administration of IPV elicits strong humoral responses conferring protection against paralytic disease, the inadequate mucosal immune responses potentially allow poliovirus to replicate in the intestines of immunized individuals with a risk to still transmit polio virus to unvaccinated individuals.^2–20,21–22^ Nonetheless, IPV is similarly effective as OPV in providing pharyngeal immunity and reducing respiratory transmission.^23^

Based on the above considerations, as the world edges closer to polio eradication, there is a strong motivation to eliminate the use of OPV vaccines and move completely to IPV-based vaccinations. However, producing IPV requires growing large quantities of live wild-type polioviruses prior to clarification, purification, and inactivation (see next section). The establishment, practice, and regulation of protocols necessary for the safe preparation of live wild-type virus is an expensive and meticulous process that significantly increases the cost of IPV and limits global production capacity. The current cost of IPV vaccines is around 15 times higher than OPV vaccines in both GAVI supported and non-supported countries.^24^ Notably, some key factors increasing the cost of IPV include the use of more viruses per human dose, additional purification steps, more QC testing, and biocontainment.^25^ Despite safety protocols, there remains a remote possibility of accidental worker infection or breach in facility containment.^26^ Especially in the post-polio eradication era, the IPV production facilities could possess the risk of accidental release that could potentially reseed wild-type poliovirus into the environment.^26^

Implementing live, attenuated Sabin strains (used in OPV) to manufacture formalin inactivated Sabin IPV (sIPV) vaccine avoids large-scale cultivation of wild-type polio strains (i.e., as currently required to produce Salk-IPV) and therefore lowers the biosafety risks.^27,28^ For example, an accidental release of the Sabin virus from manufacturing facilities would result in exposure to the same non-virulent, attenuated Sabin strains found in the extensively used OPV vaccine. Based on the above considerations, there are numerous and ongoing efforts to develop sIPV vaccines. The first sIPV containing vaccines (i.e., two quadrivalent combination vaccines (DTaP-sIPV), Quattrovac and Tetrabik) for routine immunization were locally licensed in 2012 in Japan.^29^ In a more recent development in 2020, the WHO granted prequalification status to the first sIPV vaccine (Eupolio™).^15^

### Production and testing of IPV vaccines

**Figure 2.**
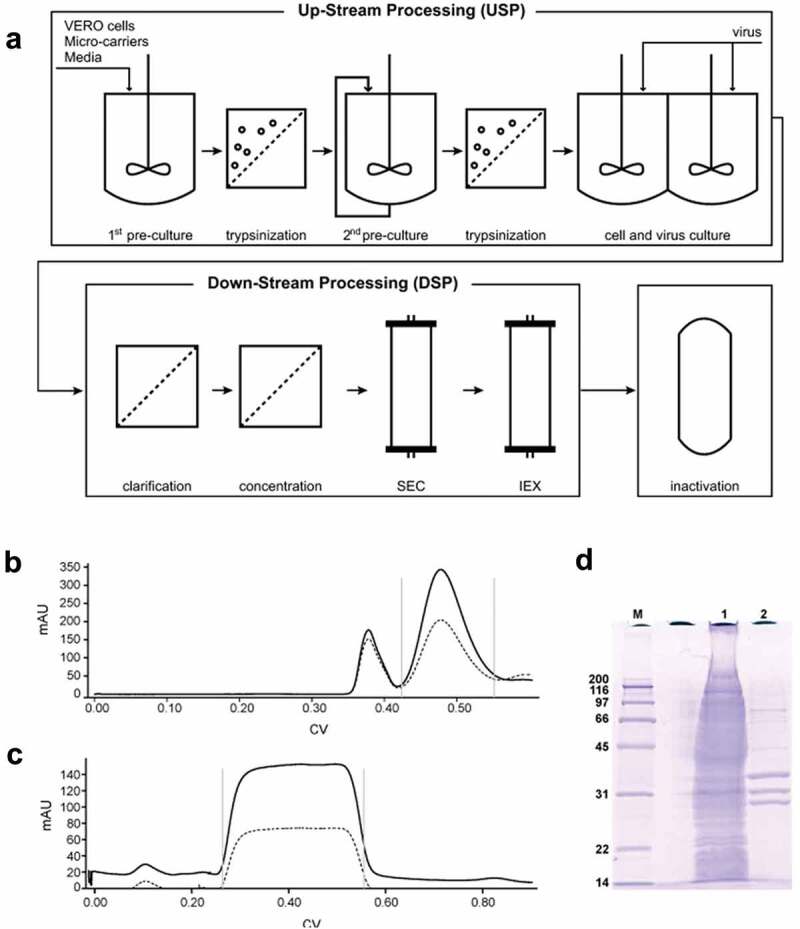
Overview of the IPV manufacturing process. (a) Upstream processing (USP) begins with two steps of increasing volume of cell growth followed by addition of seed virus for 3–4 days. The virus preparation is purified through a series of filtration steps followed by size exclusion (SEC) and ion exchange (IEC) chromatography (downstream processing, DSP). Viral inactivation is then achieved through a two-week incubation with formalin. Examples of in-process monitoring of the DSP include (b) virus separation from larger cell debris through SEC (Gray bars represent the retained volume), (c) virus elutes during IEC while impurities remain bound, and (d) SDS-PAGE gel shows the purity differences between the clarified bulk (lane 1) and the purified virus (lane 2). The bands at 33, 30, and 26 kDa represent the VP1, VP2, and VP3 protein, respectively (See Figure 1). Figures are reproduced from Bakker et al., 2011^25^ with permission from Elsevier.

Since the initial IPV production process was developed in the 1950s, it has subsequently undergone several improvements in terms of efficiency, scale-up, and enhancement of purity and yields, thus enabling cost reduction.^25^ An overview of the large-scale commercial IPV production process along with test results from the purification steps is shown in Figure 2. IPV manufacturing begins with a 15-day upstream process for culturing and scaling-up Vero (monkey kidney) cells for viral infection. The initial culture is batch-fed in a 15 L bioreactor, taking advantage of micro-carrier technology to increase cell yields. The cells are detached from the microcarriers by trypsinization and subsequently transferred to a 40 L bioreactor with additional micro-carriers. During this incubation, the cells are initially batch-fed, followed by recirculation of fresh media until the viable cell density is reached (e.g., 5 × 10^6^ cells/mL). The cells are then harvested again by another trypsinization step. The Vero cells are split into twin production vessels, grown to a viable cell density, and the serum-supplemented cell culture media are removed for serum-free media. The seed virus is added, and the cell-virus mixture is incubated for 3–4 days.^25^

The poliovirus is then purified by a series of steps called downstream processing (Figure 2). Filtration steps remove cell debris followed by size-exclusion chromatography to further remove larger sized impurities (i.e., agglomerated virus and cellular debris). The collected poliovirus peak is purified by ion-exchange chromatography. As shown by SDS-PAGE analysis, these chromatographic steps selectively remove the impurities from the clarified bulk (in lane 1) resulting in a purified viral preparation (in lane 2). The purified virus is passed through a 0.22 µm sterile filter before and during the virus inactivation step. To ensure complete inactivation of the polio virus, an incubation for 13 days at 37°C with 0.025% formalin is used.^25^ Each of the purified bulk virus bulks (Types 1, 2, 3) is subsequently diluted to their targeted doses, mixed together, and sterile-filled into stoppered glass vials to produce the final, formulated IPV dosage form (not shown).

## Analytical and preformulation characterization of polio vaccine antigens

In this section, we review analytical development and preformulation characterization studies elucidating the physicochemical and immunochemical properties of OPV, IPV, and sIPV antigens. Such studies enabled formulation development work resulting in stable, commercial dosage forms of the polio vaccines as described in subsequent sections. We first briefly cover key results with Sabin OPV (attenuated poliovirus without formalin inactivation) followed by a more detailed examination of the two formalin-inactivated antigens (IPV and sIPV).

Despite the widespread use of OPV over many decades, there have been a relatively small number of published studies to better understand the stability characteristics of the Sabin live, attenuated polio virus strains. Two major degradation pathways for the live, attenuated virus have been elucidated. First, the virus capsid proteins are sensitive to exposure to elevated temperatures. For instance, temperature-induced subtle structural changes in the viral capsid proteins can lead to loss of the conformational integrity of key epitopes on the viral capsid surface (i.e., D-antigen epitopes are critical for protective immune responses; see Figure 1). At higher temperatures, larger global structural alterations cause surface exposure of interior hydrophobic patches within the viral capsid protein, and/or protomer subunit assembly, leading to agglomeration of the virus particles.^30^ For example, a decrease in poliovirus virulence is observed at temperatures around 40°C due to thermal denaturation of the capsid protein.^31^ The addition of MgCl_2_ and D_2_O substantially increases the thermal stability of live attenuated virus (OPV) ^32^ by stabilizing the capsid protein and viral RNA.^31^ Interestingly, efforts to stabilize the capsid protein revealed that it is possible to maintain viral capsid structural integrity while still losing infectivity.^30^ The second degradation pathway is related to the stability profile of the virus’s RNA polymerase, which is greatly reduced under acidic conditions (e.g., losing almost all enzymatic activity at pH 5). One study demonstrated that OPV incubated at 45°C retains infectivity significantly better at pH 5 vs pH 7. This result was shown to correlate with the endonuclease activity of the RNA polymerase in degrading the genomic RNA necessary for viral replication.^33^

The IPV and sIPV vaccines are composed of formalin-inactivated poliovirus antigens. Formalin treatment leads to viral inactivation by alkylating a combination of amine and sulfhydryl groups within the amino acid residues of proteins, and the purine bases of nucleic acids. This alkylation reaction covalently modifies and crosslinks these functional groups within viral proteins and RNA, thereby preventing native function. A detailed analysis of formalin inactivation of poliovirus showed a reduction in the ability of viral particles (IPV) to bind to human poliovirus receptor (CD155) as compared to live poliovirus on the surface of the host cell that native polio virus interacts with during infection.^34^ Formalin treatment can also prevent the conversion from the native 160S viral particle to the structurally modified 135S particle, which is an important step for poliovirus to undergo cell entry.^34^ Finally, the formalin treatment completely prevents the infectivity of the viral RNA. These combined mechanisms result in a formalin inactivation process that prevents viral infection while leaving the key epitopes on the inactivated poliovirus particles intact to elicit a protective immune response.^34^

### In vitro and in vivo potency assays for IPV and sIPV

Batch release and stability testing of the formalin-inactivated IPV and sIPV requires the use of appropriate *in vitro* and *in vivo* potency assays since the viral particles cannot replicate. For the ELISA-based *in vitro* potency assay, the binding of conformational antibodies against the D-antigen epitopes on the surface of the IPV particles (see Figure 1) is measured. The results are defined as D-antigen units (DU) as reported by comparison to an IPV reference standard. For the *in vivo* potency assay, rat immunogenicity is measured by determining the levels of neutralizing antibodies produced. A multitude of animal models have been evaluated to assess serum neutralizing antibody titers induced by IPV vaccines including monkeys, chicks, guinea pigs, mice, and rats. Among these models, the rat model is preferred as an *in vivo* potency assay due to minimum variability between laboratories.^35^ Comparisons between sIPV and IPV samples have established that the D-antigen content per virion can differ significantly. For example, Kersten et al. (1999) demonstrated the rat immunogenicity of sIPV1 to be ~3-fold higher and sIPV2 to be ~10-fold lower than the corresponding IPV types 1 and 2, respectively, when normalized to viral mass (i.e., specific immunogenicity). The specific immunogenicity of sIPV3 and IPV3 was comparable, but D-antigenicity results of sIPV3 were ~1.5-fold lower than IPV 3. The disagreement between D-antigen antigenicity and rat immunogenicity results between sIPV and IPV types necessitate employing different assay standards for sIPV vs. IPV vaccines.^36^ Interestingly, surveillance of anti-poliovirus neutralization antibody titers in human sera can be used as a tool for monitoring vulnerability of populations to poliovirus outbreaks. For example, Arita and Iwai-Itamochi recently reported development of a high-throughput pseudo-poliovirus neutralization test (pPNT, employing noninfectious pseudovirus) that showed strong correlation with neutralizing antibody titers measured using the conventional PNT (cPNT, uses live polio virus strains), for poliovirus type 1 (OPV and sIPV), demonstrating its possible application in large-scale serosurveillance.^37^

Both the *in vitro* D-antigen ELISA and the *in vivo* rat immunogenicity assays have been used for manufacturing, quality control, and batch release of IPV vaccines. A WHO collaborative study in 1995, involving 10 laboratories, performed an analysis of six trivalent IPV vaccines (five Salk and one Sabin) using animal immunogenicity assays (in guinea pig, chick, and rat) and compared their immunogenicity with D-antigenicity content.^38,39^ Comparison of immunogenicity and antigenicity results showed an overall good correlation between the majority of preparations. These results underscore the importance and suitability of D-antigen ELISA for assessing IPV potency via quantitative assessment of D-antigen units in the IPV preparations.^39^ The IPV3 component in one preparation, however, was a noted exception showing lower immunogenicity than predicted by D-antigenicity ELISA, indicating that the above correlations are not universal. This discrepancy between the two methods for that particular sample was suggested to be due to the age of the sample, which was stored at 4°C longer than other preparations,^39^ a result consistent with comparative studies of the two potency assays with stressed IPV samples.

**Figure 3.**
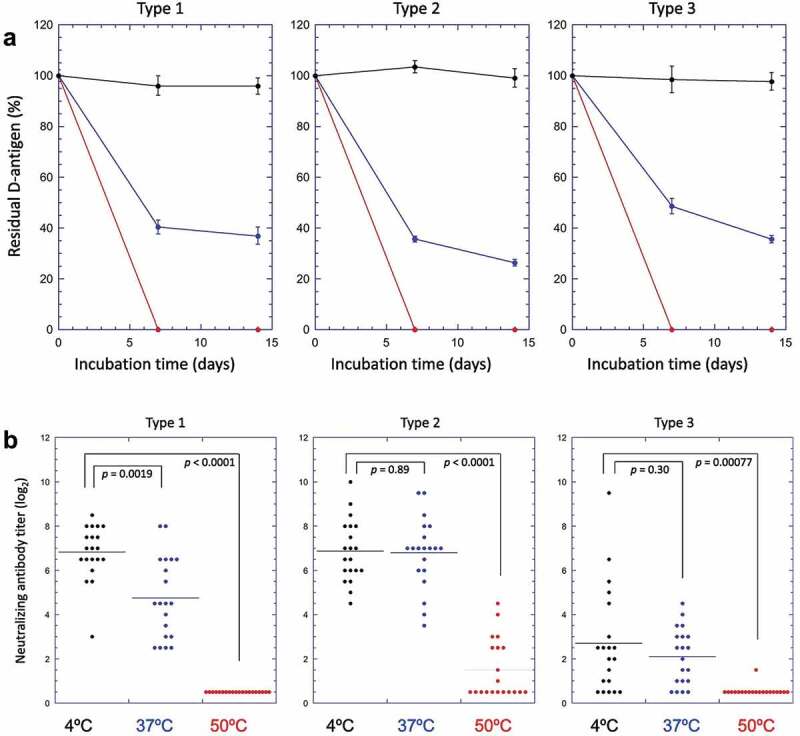
Effect of elevated temperature storage on the *in vitro* potency (D-antigenicity) and *in vivo* potency (rat immunogenicity) results for sIPV-containing combination vaccine (DTaP-sIPV). (a) D-antigenicity results of sIPV Types 1,2,3, were determined by D-antigen ELISA of samples incubated over two weeks at 4°C (black circles), 37°C (blue circles) or 50°C (red circles). The D-antigen values are expressed as a percentage of time zero sample. Error bars indicate 95% confidence intervals. (b) Rat immunogenicity results of sIPV Types 1,2,3 (neutralizing Ab titers) of samples stored at 4°C (black circles), 37°C (blue circles) or 50°C (red circles) for 1 week. Horizontal bars in each sample group indicates the average of the neutralizing antibody titers. The p-values determined by Student’s t-test were indicated in each panel. Figure presented from Murakami et. al., 2020^40^ with permission from Elsevier.

Differences in the stability profile of IPV and sIPV between the two potency assays using stressed samples (thermal and freeze-thaw) have been reported. For example, Murakami et al. (2020) and White et al. (2018) examined the effect of elevated temperatures on sIPV and freezing temperatures on IPV, respectively, as measured by *in vitro* antigenicity and *in vivo* immunogenicity.^40,41^ In both studies, stressed samples of sIPV and IPV induced robust immune responses in rat potency assays even after D-antigenicity values showed significant losses.^40,41^ In particular, Murakami et al. (2020) compared the stability of two sIPV containing combination vaccines (DTaP-sIPV) for 1 week at 50°C, 37°C, and 4°C. As shown in Figure 3, exposure to 50°C for 1 week rendered the D-antigen undetectable for all serotypes. This also corresponded to a loss in rat immunogenicity for sIPV types 1 and 3, however sIPV type 2 neutralizing antibodies were still generated. The samples stored at 37°C lost considerable D-antigenicity, with all serotypes losing greater than 50% of antigenicity when compared with the 4°C sample. Immunogenicity was less impacted by the 37°C stress condition, with some samples showing no significant losses.^40^ Hence, the remaining D-antigen content after heat treatment was sufficient to induce relatively high levels of neutralizing antibodies in rats. This study demonstrates that the *in vitro* D-antigen ELISA is more sensitive than the *in vivo* rat immunogenicity test for detecting structural alterations in sIPV viral particles induced by exposure to elevated temperatures.^40^

### Forced degradation studies with IPV and sIPV

IPV vaccines have been observed to lose potency upon exposure to a variety of environmental stresses including elevated temperatures, acidic pH, and freeze-thaw. Each of these avenues to vaccine degradation presents their own respective formulation challenges to minimize their occurrences. We present below a brief review of the characterization of IPV degradation pathways with an emphasis on the analytical assays used, the formulation “lessons-learned,” and when possible, the molecular mechanism(s) causing vaccine degradation. We first cover the results with Salk IPV vaccines followed by similarities and differences with Sabin sIPV studies.

**Figure 4.**
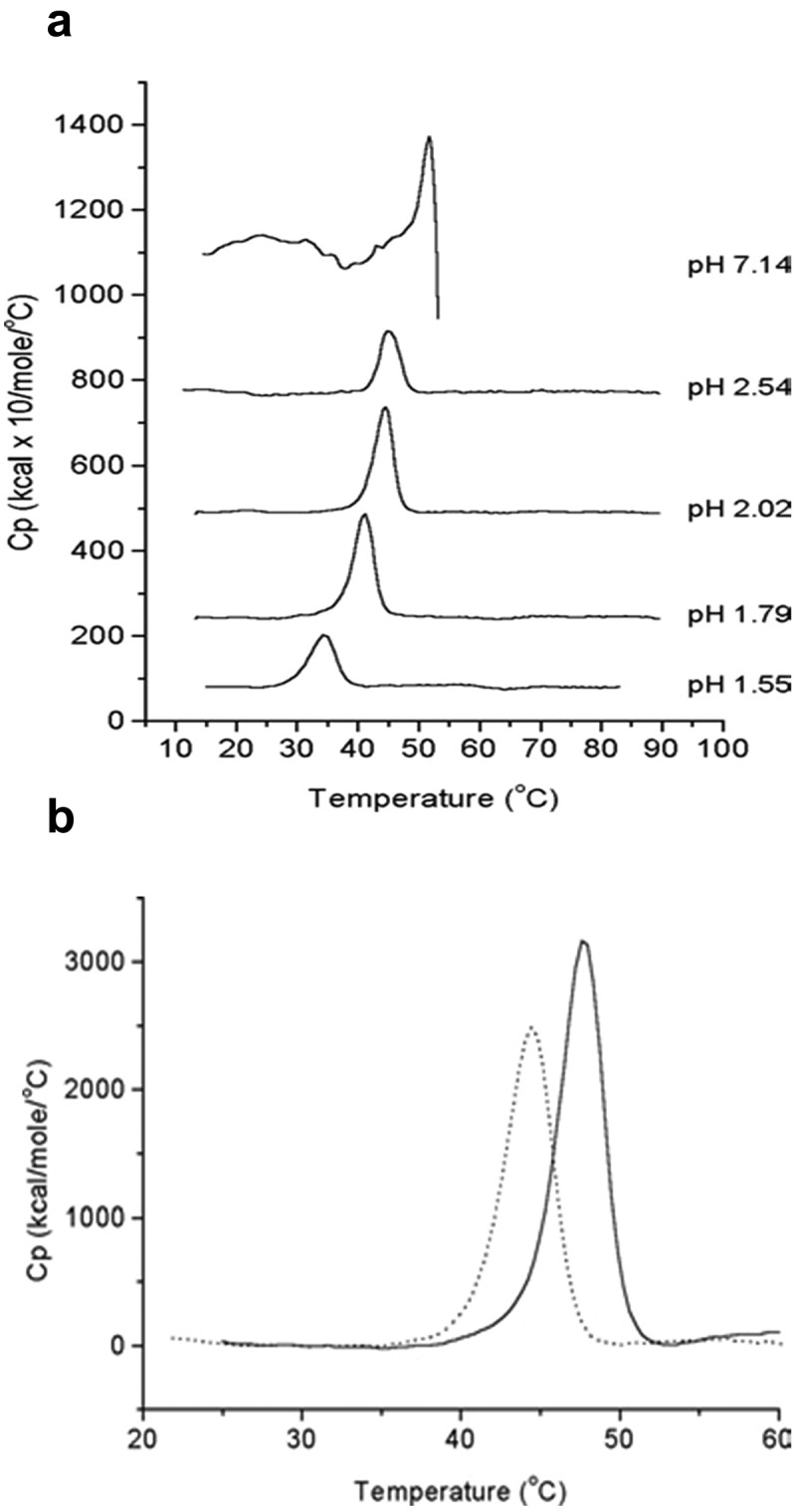
Differential scanning calorimetry (DSC) analysis of formalin-inactivated poliovirus (IPV) antigens. (a) DSC thermograms of IPV1 at neutral and acidic pH values. (b) DSC thermograms of IPV1 (dotted lines) and IPV 2 (solid lines) at pH 2 (to mimic gastric pH conditions). Figure reprinted from Krell et al., 2005^44^ with permission from Wiley.

Differential scanning calorimetry (DSC) is an informative biophysical method for measuring the overall conformational stability of a virus particle.^42,43^ As shown in Figure 4, Krell et al.^44^ evaluated IPV1 by DSC at pH ~7, showing a major transition peak at ~50°C followed by a noisy exothermic peak indicating heat-induced aggregation. At low pH values (pH ≤ 3), IPV1 aggregation was mitigated such that a better defined thermal transition was observed. At pH 2, the thermal melting temperatures (T_m_) of IPV1 and IPV2 were measured to be ~44° vs. ~48°C, respectively, indicating that IPV2 is physically more stable than IPV1 under these conditions.^44^

Using a combination of ELISA D-antigenicity and biophysical techniques, Qi et al. characterized the pH-temperature dependent stability profile of IPV serotype 3 (IPV3).^45^ Employing data visualization tools (i.e., empirical phase diagram) to compile and display stability data from intrinsic fluorescence spectroscopy (IFS), circular dichroism, and static light scattering measurements, IPV3 was shown to be physically most stable at pH 7 below 40°C. Measurements of stressed samples by IFS correlated with the ELISA D-antigenicity results, suggesting that IFS could be used as a screening assay to assess the physical stability of IPV3. Excipient screening performed by IFS identified D_2_O, sodium citrate (0.5 M), glycerol (20%), and high concentrations of saccharides (25% w/w trehalose, sucrose, and sorbitol) as having stabilizing effects for IPV3. Interestingly, the stabilizing effect of D_2_O and high saccharide concentrations for IPV3 was consistent with previously demonstrated results for the orally administered, live attenuated Sabin OPV.^30^ The stabilizing effect of high saccharide concentrations is suggested to be due to an increase in the excluded volume effect,^45^ whereas D_2_O is thought to increase the rigidity of the viral capsid thereby reducing temperature induced swelling.^32^

**Figure 5.**
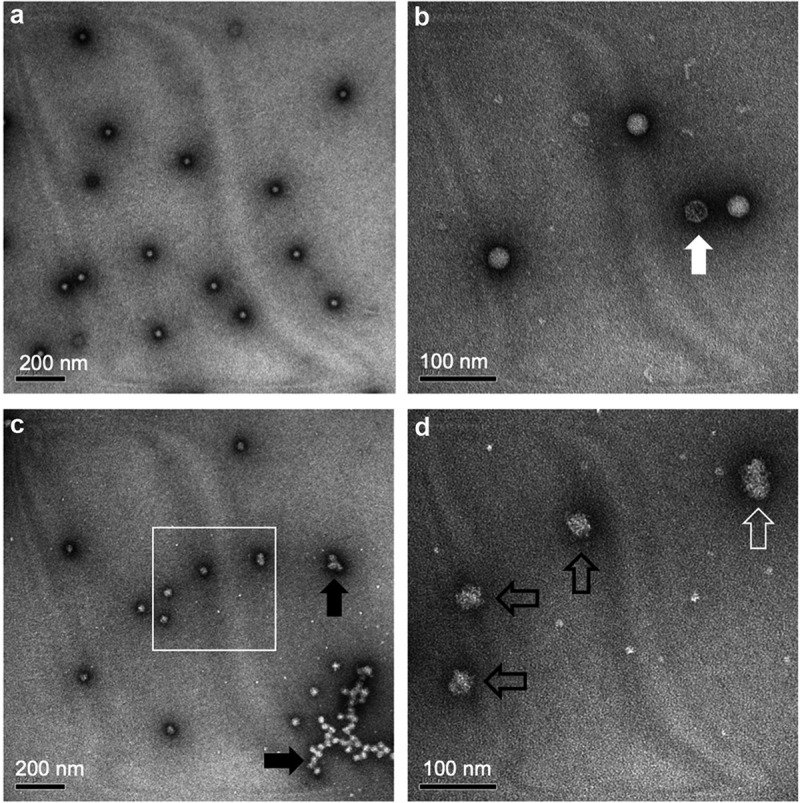
Representative transmission electron microscopy (TEM) images of sIPV 2 samples with and without low-pH exposure. (a, b) unstressed sIPV 2, and (c, d) low pH-stressed sIPV 2. Panels b and d are higher magnification images of panels a and c, respectively. Unstressed sIPV2 was characterized by a uniform distribution of spherical particles with some empty capsids (panel b, white arrow). Low pH-stressed sIPV 2 showed mixture of swollen spherical and ellipsoidal virions (panel d, black and white open arrows, respectively) as well as agglomerated viral particles (panel c, black arrows). Figure reprinted from Torisu et al., 2021^46^ with permission from Elsevier.

Overall, IPV and sIPV antigens display similar physicochemical properties and stability profiles; for instance, both are less stable under acidic pH conditions.^46^ During the sIPV manufacturing process, prior to purification by cation exchange chromatography (see Figure 2), the solution pH is lowered to pH 4.0. Torisu et al. (2021) determined the physical properties of sIPV with and without low pH exposure.^46^ First, the morphology and size of sIPV viral particles were visualized using TEM. For the unstressed sample, a nearly uniform distribution of spherical sIPV viral particles was observed (Figure 5, Panel A, B). Upon low pH exposure, a mixture of swollen and aggregated viral particles was noted (Figure 5, Panel C, D). By utilizing a panel of size and aggregation assays, in tandem with the D-antigenicity assay, Torisu et al. also elucidated a mechanism by which low pH induces swollen virions and aggregated virions, which leads to a loss of D-antigenicity.^46^ As summarized in Table 1, exposure to acidic pH had a significant effect on the size of sIPV viral particles in solution as measured by a combination of biophysical techniques including dynamic light scattering (DLS), asymmetrical flow field-low fractionation coupled to multi-angle laser light scattering (AF4-MALS), sedimentation velocity analytical ultracentrifugation (SV-AUC).^46^ Significantly larger particle size at pH 2 (~50 nm) vs. pH 7 (~30 nm) as measured by DLS. AF4-MALS and SV-AUC analysis of unstressed sIPV determined that nearly 100% of the viral particles were eluted in a single peak, while low pH stressed samples displayed a biphasic elution profile with less than 50% of the viral particles in the main peak along with an additional peak indicating that some virions had agglomerated, a result consistent with TEM images described above. In summary, prolonged exposure to low pH conditions should be avoided during manufacturing to minimize the loss of D-antigenicity due to irreversible structural changes (swelling) and agglomeration of sIPV virus particles.

**Table 1.** Summary of the effects of low pH exposure on the size and aggregation of sIPV 2 as measured by different biophysical techniques. Compared to unstressed samples, the low pH stressed sIPV 2 displayed increased particle size while maintaining the same molar mass indicating swelling of the sIPV 2 viral particles. The reduction of the main peak areas as measured by AF4-MALS and SV-AUC indicates the formation of larger agglomerates of viral particles. The TEM data visually showing these effects are also displayed in Figure 5. Figure reproduced from Torisu et al., 2021 ^46^ with permission from Elsevier.

		Diameter (nm)	Content (%)	Molar Mass (10^6^ g/mol)	*s*_20,w_	Frictional Ratio
Unstressed IPV	AF4-MALS	26.3 ± 1.3	99.4 ± 0.2	8.28 ± 0.15	–	–
	SV-AUC	24.4 ± 0.0	82.3 ± 1.1	–	156 ± 0.1	1.22
	DLS	29.5 ± 2.1	–	–	–	–
	TEM	24 ± 3	–	–	–	–
Low pH stressed IPV	AF4-MALS	41.5 ± 1.6	47.3 ± 0.6	8.47 ± 0.16	–	–
	SV-AUC	–	43.9 ± 3.5	–	95 ± 0.5	–
	DLS	50.6 ± 4.8	–	–	–	–
	TEM	33 ± 14	–	–	–	–

Other sIPV degradation studies based on exposures encountered during manufacturing include the impact of phenol red, freeze–thaw and different containers. Phenol red, a colorimetric dye used in tissue culture media to monitor pH during fermentation, had a significant stabilizing impact on the sIPV2 at lower pH.^47^ Freezing, caused either intentionally by storage at −20°C or inadvertently by improper cold storage, resulted in a significant reduction in D-antigen content.^48^ For example, the D-antigen content measured in vaccine samples stored at −20°C for 1-day, or 1-week, or after mimicking improper cold storage for 3 freeze–thaw cycles was significantly reduced. These same samples were, however, still capable of eliciting seroprotection in mice after three doses of immunization without a significant difference in geometric mean antibody titers.^48^ It has also been reported that sIPV serotypes 1 and 2 adsorb to standard pharmaceutical glass vials, which can be prevented by using siliconized glass.^36^ Finally, Westdijk et al. have recently demonstrated the advantages of combining immunochemical and biophysical techniques as a rapid quality assessment tool to support and monitor the structural integrity and stability of sIPV antigens during the manufacturing process.^49^

## Commercially available IPV and sIPV vaccine formulations

Currently, there are nine stand-alone commercial IPV vaccines (Table 2) and six commercially available IPV containing combination vaccines (Table 3) as listed by the US FDA and WHO prequalification websites.^50–52^ These two tables do not include additional IPV/sIPV containing vaccines produced by local manufacturers. As shown in Table 2, most of the standalone IPV vaccines are unadjuvanted and contain 40, 8, and 32 DU for IPV types 1, 2, and 3, respectively. The two exceptions include the most recently introduced IPV vaccines, namely Picovax® and Eupolio® (last two entries in Table 2). Picovax® is the first adjuvanted IPV vaccine developed by AJ Vaccines that recently received WHO prequalification. Due to the dose-sparing effects of 0.5 mg aluminum hydroxide adjuvant Alhydrogel®, the IPV dose in Picovax® is ~10 times lower than the standard IPV vaccines. Eupolio® is the first Sabin sIPV vaccine to obtain WHO prequalification containing a notably lower dose of 5, 8, and 16 SDU for sIPV1, 2, and 3.^53^This difference in sIPV dose can be attributed to the differences in antigenicity and immunogenicity profiles and reference standards between IPV and sIPV ^36^ as described above.

**Table 2.** Formulation composition, type of dosage form, and stability summary of commercially available trivalent IPV vaccines. The table summarizes stand-alone IPV vaccines approved by the US FDA and/or prequalified by the World Health Organization (WHO); the composition is based on publicly available information ^50–52^. The table does not include IPV and sIPV produced by local manufacturers. One aluminum adjuvanted IPV (Picovax™) vaccine and one sIPV (Eupolio™) vaccine are currently available. IPV- Inactivated polio vaccine, sIPV - Inactivated Sabin polio vaccine, Al(OH)_3_ - aluminum hydroxide, 2-PE - 2-phenoxyethanol, PS-80 - polysorbate 80, *M*-199 - medium *M*-199 or its modification, IM - intramuscular, SC - subcutaneous, ND - no designation.

Trade Name (Manufacturer)	Active ingredients/dose	Inactive ingredients/dose	Aluminum content/dose	Dosage form	Route of administration	Dose (mL)	Nos. of doses per vial	VVM designation	Shelf life at 2-8°C (Months)	Status
IMOVAX POLIO (Sanofi Pasteur SA)	IPV1: 40DU, IPV2: 8DU, IPV3: 32DU	M-199, 2-PE (≤1.0%), Formaldehyde (≤0.02%)	None	Liquid	SC	0.5	10	7	36	WHO-PQ 2005
Poliomyelitis Vaccine (Serum Institute of India Pvt. Ltd.)	IPV1: 40DU, IPV2: 8DU, IPV3: 32DU	2-PE (2.5 mg), Formaldehyde (12.5 µg)	None	Liquid	IM or SC	0.5	1, 2, 5, 10	7	36	WHO-PQ 2016, 2019
Poliomyelitis vaccine (Bilthoven Biologicals B.V.)	IPV1: 40DU, IPV2: 8DU, IPV3: 32DU	M-199 (0.1 ml), Formaldehyde (12.5ug), 2-PE (2.5 mg), disodium hydrogen phosphate dehydrate, potassium chloride, sodium chloride, potassium dihydrogen phosphate, PS-80, calcium chloride, phenol red	None	Liquid	IM or SC	0.5	1, 5	7	36	WHO-PQ 2010, 2014
Poliorix (GlaxoSmithKline Biologicals SA)	IPV1: 40DU, IPV2: 8DU, IPV3: 32DU	2-PE (5 mg), M-199, Formaldehyde, PS-80	None	Liquid	IM	0.5	1, 2	14	36	WHO-PQ 2010
ShanIPV (Sanofi Healthcare India Private Limited)	IPV1: 40DU, IPV2: 8DU, IPV3: 32DU	M-199, 2-PE (2.5 uL), Formaldehyde (12.5 µg), Ethanol	None	Liquid	IM or SC	0.5	5	11	36	WHO-PQ 2018
IPOL (Sanofi Pasteur SA)	IPV1: 40DU, IPV2: 8DU, IPV3: 32DU	M-199, 2- PE (0.5%), Formaldehyde (0.02%),	None	Liquid	IM or SC	0.5	10	ND	36	US FDA 2012
IPV Vaccine SSI (AJ Vaccines A/S)	IPV1: 40DU, IPV2: 8DU, IPV3: 32DU	M-199 (to 0.5 mL)	None	Liquid	IM or SC	0.5	1	7	36	WHO-PQ 2010
Picovax (AJ Vaccines A/S)	IPV1: 3.2DU, IPV2: 0.88DU, IPV3: 3.1DU	M-199, Sodium hydroxide, Sodium phosphate monobasic, monohydrate, Sodium chloride, 2-PE	Al(OH)_3_ Al: 0.5 mg	Liquid	IM	0.5	5	7	24	WHO-PQ 2020
Eupolio Inj. (LG Chem Ltd)	sIPV1: 5DU, sIPV2: 8DU, sIPV3: 16DU	2-PE (2.5 mg), Formalin (17.5 µg)	None	Liquid	IM	0.5	1, 5	7	30	WHO-PQ 2020, 2021

**Table 3.** Formulation composition, type of dosage form, and stability summary of commercially available trivalent IPV containing combination vaccines. The table summarizes pediatric combination vaccines containing trivalent IPV approved by the US FDA and/or prequalified by the World Health Organization (WHO). The composition is based on publicly available information ^50–52^. The table does not include combination vaccines containing IPV and sIPV produced by local manufacturers. IPV – Inactivated polio vaccine, sIPV - Inactivated Sabin polio vaccine, Al(OH)_3_ - aluminum hydroxide, AlPO_4_ - aluminum phosphate, IM - intramuscular, PS-80- polysorbate 8, ND – no designation, NA – not available.

Trade Name	Active ingredients/dose	Inactive ingredients/dose	Aluminum content/dose	Dosage form	Route of administration	Dose (mL)	Number of doses per vial	VVM designation	Shelf life at 2-8°C (Months)	Status
Hexaxim (Sanofi Pasteur SA)	DTaP-Hib-HepB- IPV IPV1: 40DU, IPV2: 8DU, IPV3: 32DU	Disodium hydrogen phosphate (1.52 mg), Potassium dihydrogen phosphate (1.55 mg), Trometamol (0.15 mg), Saccharose (10.6 mg), Essential amino acids (1.115 mg)	Al(OH)_3_ Al: 0.6 mg	Liquid	IM	0.5	1	7	36	WHO-PQ 2014
Vaxelis (MSP Vaccine company)	DTaP-Hib-HepB- IPV IPV1: 29DU, IPV2: 7DU, IPV3: 26DU	PS-80 (<0.0056%)	Mixed Aluminum Al: 0.32 mg	Liquid	IM	0.5	1	ND	42	US FDA 2018
Pentacel (Sanofi Pasteur LTD)	DTaP-Hib-IPV IPV1: 40DU, IPV2: 8DU, IPV3: 32DU	PS-80 (~10 ppm), Sucrose (42.5 mg)	AlPO_4_ Al: 0.33 mg	Suspension (DTaP-IPV is liquid, Hib is lyophilized)	IM	0.5		ND	NA	US FDA 2008
Pediarix (GlaxoSmithKline Biologicals)	DTaP-HepB- IPV IPV1: 40DU, IPV2: 8DU, IPV3: 32DU	Sodium chloride (4.5 mg), PS 80 (≤100 µg)	Mixed aluminum Al: 0.85 mg	Liquid	IM	0.5	1	ND	NA	US FDA 2002
Quadracel (Sanofi Pasteur LTD)	DTaP-IPV IPV1: 40DU, IPV2: 8DU, IPV3: 32DU	PS-80 (~10 ppm)	AlPO_4_ Al: 0.33 mg	Liquid	IM	0.5	1	ND	NA	US FDA 2015
Kinrix (GlaxoSmithKline Biologicals)	DTaP-IPV IPV1: 40DU, IPV2: 8DU, IPV3: 32DU	Sodium chloride (4.5 mg), PS-80 (≤100 µg)	Al(OH)_3_ Al: 0.5 mg	Liquid	IM	0.5	1	ND	36	US FDA 2008

A summary of combination pediatric vaccines containing IPV antigens as listed by the US FDA and WHO prequalification websites is displayed in Table 3.^50–52^ Each of these IPV-containing combination vaccines also contain diphtheria, tetanus, and acellular pertussis antigens, while some newer ones also include Hepatitis B and *Haemophilus influenzae* type b. Notably, none of these US FDA and WHO prequalified IPV containing combination vaccines contain whole-cell pertussis (wP) and none are available in multidose formats (i.e., single-dose presentations contain one vaccine dose per vial, while multi-dose typically contain 2 to 10 vaccine doses in single vial to reduce cost and improve vaccine coverage;^54^ see next section). The reported shelf-life values of the commercially available IPV containing combination vaccines are 3 to 3.5 years at 2–8°C, although this information has not been disclosed for some products (Table 3).

In contrast, for the standalone IPV vaccine, much more stability information is publicly available (Table 2). Most standalone IPV vaccines have a shelf life of 3 years when stored at 2–8°C and need to be transported in the cold chain. IPV storage stability data over a 20-year period in the absence of preservatives has been reported.^55^ Immunogenicity testing in guinea pigs showed IPV1 to be the least stable, significantly losing potency after 2 years, while IPV2 and IPV3 were stable when stored at 4°C over a 20-year period.^55^ The authors also tested the immunogenicity in guinea pigs with IPV in combination with Al(OH)_3_-bound diphtheria and tetanus toxoids when stored at 4°C. The IPV2 and IPV3 serotypes were stable over the 10-year period. Finally, the authors stored a trivalent IPV at 4°, 24°, and 32°C for 20 days and assessed antigenicity by ELISA and immunogenicity in guinea pigs, rats, and mice. The IPV2 serotype remained stable at all temperatures, while a significant loss in IPV3 D-antigenicity values in the 32°C sample was observed. This, however, did not translate into loss of immunogenicity in animals. For the IPV1 serotype, a significant loss in D-antigenicity was observed when stored at 24°C, and a complete loss when stored at 32°C. The immunogenicity of IPV1 serotype in animals, however, showed a less pronounced temperature-dependent loss.^55^

Since IPV is freeze sensitive, it is also important that the vials do not encounter freezing temperatures during shipping and storage in the vaccine cold chain.^56^ White et al. (2018) assessed the freeze sensitivity of commercial IPV vaccines, namely VeroPol (single dose) and IPOL (multi-dose).^41^ The freezing of VeroPol at −20°C for 7 days had no notable effect on ELISA D-antigenicity and immunogenicity as measured using *in vivo* rat potency assay. Freezing IPOL under similar conditions, however, showed clear trends of loss in ELISA D-antigen contents for all three IPV types, yet no effect in immunogenicity was noted using the rat potency assay.

## Low-cost and next-generation IPV vaccine formulations

As outlined above, replacing Salk IPV with Sabin sIPV antigens is anticipated to provide significant cost savings due to reduced manufacturing costs.^57^ Nonetheless, for older vaccines such as IPV that have been produced for decades and are in the later stages of their product lifecycle, drug product costs (i.e., formulation, dosage form, fill-finish manufacturing, distribution/shipping, and administration) remain one of the biggest cost drivers.^58^ In this section, we explore IPV vaccine formulation strategies for reducing costs and/or improving vaccine compliance, with the goal of increasing vaccine coverage to eradicate polio worldwide. Shorter-term, new vaccine dosage forms can lower costs, including employing multi-dose formulations (more vaccine doses per vial), and in combination vaccines (more vaccine antigens per vial). Formulation approaches to increase storage stability at elevated (and freezing) temperatures can also lower costs by simplifying the vaccine cold-chain requirements. Longer-term, more novel IPV formulation strategies include dose-sparing, enhanced immunogenicity, and improved ease-of-use via conventional and novel vaccine adjuvants and delivery systems. Such approaches offer the potential for improved vaccine coverage in LMICs. Implementation of these shorter- and longer-term formulation strategies, alone and eventually in combination, has great potential to enable low-cost, patient-friendly IPV vaccines targeted for use in LMICs for polio eradication.

### *Multi-dose*, *combination, and thermostable IPV formulations*

Although single-dose vials and prefilled syringes are a convenient method to deliver intramuscularly injected IPV vaccines, they cost more to manufacture, take up more space in the vaccine cold chain, and create more medical waste compared to multi-dose formulations.^54,59^ Multi-dose formulations allow for multiple vaccine doses to be obtained from a single vial by inserting multiple needles into the same vial. To prevent potential bacterial contamination, multi-dose IPV vaccines are formulated with antimicrobial preservatives (APs). The formulation challenge in terms of multidose vaccines is to identify sufficient amounts of APs to prevent microbial growth while maintaining good long-term storage stability of the antigens.

For standalone IPV vaccines, 2-phenoxyethanol has been added as an AP to prepare commercially available multidose formulations (Table 2).^60^ The commercially available standalone IPV multi-dose presentations are required to follow a 4-point WHO multi-dose vial policy (MDVP) before administration,^61^ i.e., the vaccine should be (1) WHO prequalified at the time of administration, (2) approved for use up to 28 days after opening the vial, (3) unexpired, and (4) continued to be stored at a manufacturer-recommended temperature with vaccine vial monitors (VVMs) not past the discard point. Unfortunately, another commonly used AP in vaccines, thimerosal, destabilizes IPV ^62^ and cannot be used for multi-dose IPV formulations.

The deleterious effect of thimerosal on poliovirus vaccines was initially reported in 1956.^63^ Sawyer et al.^64^ subsequently showed that the stability of IPV stored at 4°C was reduced when preserved with 0.01% or 0.005% thimerosal compared to IPV preserved with 2-phenoxyethanol. The reduced stability was observed for all three serotypes; however, IPV1 was more sensitive to thimerosal-induced destabilization than IPV2 or IPV3. The inclusion of DTP was not observed to induce further destabilization of the antigenicity compared to IPV stored with thimerosal. The reduced storage stability measured by antigenicity was paralleled to a reduced potency in mice as measured by antibody production after immunization. The authors also reported that clinical trials in children administering IPV and DTP using a dual-channel syringe also resulted in reduced antibody titers compared to coadministration, a result suggesting that thimerosal and IPV interacted long enough during the administration procedure with the dual-chamber syringe to destabilize the vaccine.

The incompatibility of IPV with thimerosal has complicated efforts to add IPV to the trivalent DTP (DTwP) ^62^ and pentavalent (DTwP-Hib-Hep B)^64^ combination vaccines that contain inactivated whole-cell pertussis antigen (wP), since wP is manufactured with thimerosal as an inactivating agent. There are two types of pertussis vaccine antigens, acellular (aP) and whole cell (wP). Although both antigens provide overall comparable immunogenicity, wP is much less expensive to produce than aP (requiring manufacturing of up to five different individual antigens), making it preferable for use in LMICs.^65^ The development of combination pediatric vaccines containing both IPV and wP is thus a challenging formulation goal.^62,66,67^ Kraan et al. observed immediate temperature-dependent loss of efficacy upon resuspension of lyophilized IPV, with pentavalent vaccine suggesting that the thimerosal-induced destabilization occurs too quickly for resuspension to be a feasible option.^64^ Interestingly, thimerosal scavenger L-cysteine was able to protect IPV from thimerosal-induced destabilization,^64^ but has not been implemented, likely due to its effects on antimicrobial effectiveness.

The addition of IPV to pediatric combination vaccines containing wP requires a new manufacturing process for wP without the use of thimerosal. Such efforts are ongoing at developing country vaccine manufacturers (DCVMs). Interestingly, EasySix® (Panacea Biotec, India), locally licensed for use in India, contains both IPV and wP in a combination vaccine.^68^ EasySix® is a hexavalent vaccine (DTwP-Hib-Hep B-IPV) adjuvanted to 1.25 mg Aluminum in the form of aluminum phosphate gel. The use of 2-PE as a preservative offers a multidose format. Additional vaccine candidates containing both wP and IPV in combination vaccines are in the pipeline including SHAN6^TM^ (Sanofi Health Care India) as well as candidates from other DCVMs (e.g., Serum Institute of India and LG Chem Ltd.).^69–71^ Development of IPV containing pediatric combination vaccines will undoubtedly play a crucial role in providing polio immunity worldwide with a low-cost multi-dose, combination vaccine formulation of IPV.

Thermostable IPV vaccines could also significantly reduce costs and permit long-term stockpiling of IPV vaccines. Exposure to elevated temperatures plays a major role in the instability of vaccines due to gaps in the vaccine cold chains in LMICs. The vaccine cold-chain infrastructure costs a substantial amount of money to implement and maintain. One formulation strategy to improve thermostability of vaccines is lyophilization. This sublimation-based drying process results in a dried cake containing the vaccine drug product with small amounts of residual moisture (~1–3% water, w/w). Many lyophilized pharmaceutical drug products (small molecule and protein-based drugs as well as vaccines) are more stable and less sensitive to temperature changes in this dried state.^72^

Recent studies have explored the possibility of formulating a lyophilized IPV vaccine. One such study found that a combination of sorbitol, monosodium glutamate, and magnesium chloride significantly stabilized the trivalent IPV strain during the lyophilization process and subsequent storage.^73^ A second such study found that submolar concentrations of urea could stabilize IPV during lyophilization in combination with sucrose. The authors noted that MgCl_2_ and urea both cause chaotropic effects, which may stabilize the viral particles by preventing agglomeration.^74^ Another study in 2018 using inactivated Sabin polio vaccines identified a lyophilized formulation of sIPV containing histidine, mannitol, MgSO_4_, sorbitol, and pluronic F68.^75^ The development of a stable, lyophilized IPV could be invaluable for eliminating cold-chain requirements; however, such advantages of improved stability need to be considered in the context that freeze-drying is more costly to manufacture, and there is limited worldwide production capacity.^76^

### IPV vaccines formulated with aluminum adjuvants

Aluminum-salts are the most commonly used and lowest cost vaccine adjuvants. They have a long history of increasing immune responses to vaccine antigens with minimal side effects. Examples of currently used aluminum adjuvants containing vaccines include inactivated viruses (e.g., inactivated Hepatitis A), purified antigens from bacteria (e.g., diphtheria, tetanus, and acellular pertussis), and recombinant viral antigens (e.g., Hepatitis B and HPV).^77^ Although standalone conventional IPV vaccines have been formulated without adjuvants, recent clinical trials have demonstrated that adjuvants can produce non-inferior immune responses with less IPV, suggesting that adjuvants can enable lower-dose and less expensive IPV and sIPV vaccines.^78^

For standalone IPV vaccines with aluminum adjuvants, preclinical models have established dose-sparing effects,^79^ and clinical trials with aluminum-adjuvanted IPV and sIPV have more recently demonstrated their safety and immunogenicity.^80,81^ In December 2020, the WHO awarded prequalification status to Picovax®, an aluminum adjuvanted IPV vaccine containing one-tenth of the typical IPV dose.^53^ Interestingly, there have been reports of IPV use with aluminum adjuvants for many decades, yet their implementation has only recently occurred. For example, aluminum phosphate adjuvant was used to bind and concentrate polio antigens and appeared to improve thermostability.^82^ The method was incapable of eluting more than 10–20% of the antigen content, however, suggesting that polio antigens bind strongly to aluminum adjuvants.^83^ Early animal immunogenicity studies in the 1960s evaluating IPV with aluminum adjuvants in guinea pigs demonstrated that IPV bound to aluminum phosphate induced higher antibody titers than unadjuvanted IPV. A study in rhesus monkeys assessed aluminum hydroxide as an IPV adjuvant for a Type 1 monovalent vaccine. The adjuvanted vaccine elicited significantly higher antibody titers, and fewer monkeys (12%) developed paralytic polio when challenged with a combination of live poliovirus and immunosuppressive compounds when compared to the unadjuvanted group (79%). These results were later confirmed using a trivalent IPV.^84^

IPV has been successfully added to commercially available pediatric combination vaccines that contain aluminum adjuvants as summarized in the previous section (Table 3). For example, a 2001 clinical study compared a stand-alone IPV vs. a combination IPV vaccine containing diphtheria, tetanus, acellular pertussis, hepatitis B antigens, and both aluminum hydroxide and aluminum phosphate adjuvants. The study found the combination vaccine elicited significantly higher antibody titers for IPV serotypes 1 and 3, while IPV type 2 antibodies were higher but did not reach significance. This trial did not find significant differences in local side effects upon administration.^84^ Two more recent studies have compared stand-alone IPV with a diphtheria, tetanus, acellular pertussis, *Haemophilus influenzae*, and IPV combination. Aluminum hydroxide containing Pentaxim® increased erythema and swelling incidents, while also eliciting significantly higher antibody titers for each polio serotype. The aluminum phosphate containing Pentacel® elicited a similar immune response to the stand-alone IPV without any significant increase in side effects. The general increase in IPV antibody titers seen across its use in most pediatric combination vaccines suggests it may be possible to use lower IPV doses in these combination vaccines, but currently approved combinations, such as Hexaxim®, use the same dose as standalone IPV.^84^ Future research using aluminum adjuvants could lead to lower-dose IPV in pediatric combination vaccines as well as with sIPV vaccines.

### IPV formulated with novel adjuvants

In the past few decades, new adjuvants have been extensively evaluated in preclinical studies with IPV antigens to achieve dose-sparing and/or enhanced mucosal immunity effects (Table 4). These preclinical studies demonstrate the potential for improving IPV-induced immunity and cost reduction by dose sparing. We first review the use of novel adjuvants with both IPV and sIPV antigens formulated with emulsion/liposome-based adjuvants and then in formulations with various immunostimulatory molecules.

**Table 4.** Examples of preclinical studies and key findings with IPV and sIPV vaccines formulated with novel adjuvants. IM – intramuscular, ID – intradermal, SC – subcutaneous, SL – sublingual, in – intranasal.

Adjuvant	IPV dose	Immunization model	Key findings	Reference
Oil-in-water emulsions Emulsion 1 (EM1) and stable emulsion (SE)	IPV1: 16.2–0.02 DU IPV2: 16.2–0.02 DU IPV3: 16.2–0.02 DU	IM injection in female outbred Wistar rats	Oil-in-water emulsion adjuvanted trivalent IPV are dose-sparing as compared to unadjuvanted control IPV as determined using neutralizing antibody titers Dose sparing	^85^
CAF01 (dimethyldioctadecylammonium & trehalose 6,69-dibehenate)	IPV1: 2, 20 DU IPV2: 2, 20 DU IPV3: 2, 20 DU	IM and ID immunization in CB6F1/C57BL/6×Balb/c mice	IPV adjuvanted with CAF01 increased systemic immunity as measured by serum neutralization antibody titers CAF01 also enhanced the kinetics and magnitude of cellular and humoral response specific to IPV Dose sparing	^86^
GVI3000 (Alphavirus-based adjuvant)	IPV1: 0.8 DU IPV2: 0.2 DU IPV3: 0.6 DU	IM injection in Balb/c mice Potency test in outbred Rivm:TOXrats	The adjuvanted IPV vaccine increased systemic IgG, mucosal IgG, and mucosal IgA antibody responses to IPV (types 1, 2, and 3) Enhanced potency as measured serum neutralizing antibodies in rats Dose sparing	^87^
dmLT (double mutant heat-labile enterotoxin from E. coli LT(R192 G/L211A))	IPV1: 0.5, 1, 5, 10, 15 DU IPV2: 0.5, 1, 5, 10, 15 DU IPV3: 0.5, 1, 5, 10, 15 DU	ID or IM injection in female BALB/c	Unadjuvanted ID delivery was inferior to IM delivery dose reduction but led to improved mucosal immunity Immunization with dmLT adjuvanted IPV promoted serum anti-PV neutralizing antibodies by either IM or ID delivery Dose sparing	^88^
dmLT in thermoresponsive gel (TRG) delivery system	IPV1: 1.34, 6.5 DU IPV2: 0.3, 1.5 DU IPV3: 1.1, 5.5 DU	SL in TRG or IM phosphate buffered saline in BALB/cJ mice	SL delivery led to production of both mucosal and serum antibodies including IgA IM delivery produced only serum neutralizing and binding Ig but no detectable IgA	^89^
CpG oligodeoxynucleotides (CpG-ODN)	sIPV1: 2.5, 0.6, 0.2 DU sIPV2: 10, 2.5, 0.6 DU sIPV3: 2.5, 0.6, 0.2 DU	IM injection of sIPV in Balb/c mice	sIPV with CpG-ODN alone or in combination with alum enhanced both humoral and cell mediated immune responses Dose sparing	^90^
Chitosan (chitosan glutamate and chitosan sulfate micro/nanoparticles)	sIPV1: 4.0, 0.40, 0.04 DU sIPV2: 0.8, 0.08, 0.01 DU sIPV3: 3.2, 0.32, 0.03 DU	IM injection in female Balb/c mice	sIPV with chitosan glutamate or chitosan sulfate micro/nanoparticles elicited significantly higher immunogenicity to sIPV (type 1, 2, 3) vs. unadjuvanted sIPV control Dose sparing	^91^
1,25-Dihydroxyvitamin D3 (DHVD3)	sIPV1: 40 DU sIPV2: 8 DU sIPV3: 32 DU	IP injection in three lines of male and female mice, CD-1, Balb/c, NIH(S)	DHVD3 co-administered with monovalent IPV 1, 2 or 3 enhanced systemic and mucosal immunity Dose sparing	^92^
*Quillaja brasiliensis* or Quil-A Saponins	Sabin IPV 150 uL IPV1	SC or hind neck immunization in female Swiss mice of CF-1 breed	Saponins from *Q. brasiliensis* and Quil-A elicited significantly higher cellular, humoral, and mucosal immune responses to IPV type 1 Dose sparing	^93^
Cholera toxin	sIPV1: 10 DU sIPV2: 16 DU sIPV3: 32 DU	SL, IN, IM in BALB/cOlaHsd mice	Cholera toxin adjuvanted sIPV delivered via SL and IN routes led to both mucosal and systemic immunity Mucosal delivery only led to systemic immunity	^94^

Baldwin et al. (2011) demonstrated dose sparing effects of trivalent sIPV vaccine in rats using two different oil-in-water emulsion adjuvants. One formulation was a MF59®-like emulsion (squalene, Tween® 80, Span® 85 in citrate buffer, pH ∼6) and the other was called a stable emulsion (squalene, glycerol, egg phosphatidylcholine, Pluronic® F68 in ammonium phosphate buffer, pH ∼5.5).^85^ Significant increases in antibody titers against IPV type 2 vs. unadjuvanted IPV control were observed in both formulations. Dietrich et al. (2014) studied dose sparing effect by formulating IPV in liposomal CAF01 adjuvant.^86^ IPV admixed with CAF01 demonstrated enhanced serum neutralization antibody titers in mice as compared to unadjuvanted control. Further, simultaneous immunizations at an intradermal (ID) and intramuscular (IM) site were useful in generating the desired intestinal immunity against IPV.^86^

For enhanced mucosal immunogenicity effects with IPV antigens formulated with immunostimulatory molecules, the use of double mutant heat-labile enterotoxin from *E. coli* (dmLT) has gained considerable attention. The dmLT adjuvant contains two mutations in *Escherichia coli* toxin for reduction of toxicity while retaining the adjuvant properties. Norton et al. (2015) demonstrated immunization of mice via either ID or IM delivery with trivalent IPV formulated with dmLT promoted high levels of neutralizing antibodies (leading to a ≥5-fold dose sparing effect) and enhancement of mucosal immunity (high levels of fecal and intestinal anti-PV IgA) vs. unadjuvanted IPV control.^88^ White et al. (2014) ^89^ examined the sublingual administration of trivalent IPV vaccines using a combination of dmLT adjuvant with thermoresponsive gel (TRG). The TRG delivery system becomes viscous upon contact with the mucosal surface and helps to retain the formulation (sIPV + dmLT) at the site of delivery. Both mucosal and serum antibodies including IgA were observed in mice immunized using the dmLT-TRG delivery system.^89^ Another example is IPV formulated with alphavirus-based adjuvant (GVI3000).^87^ The IM injected adjuvanted IPV-GVI3000 vaccine enhanced systemic IgG, mucosal IgG, and mucosal IgA immunoglobulin levels to IPV (types 1, 2 and 3) in mice and serum neutralizing antibodies in rats.^87^

Other immunostimulating molecules have been evaluated as adjuvants with IPV antigens including oligodeoxynucleotides (CpG), chitosan, 1,25-Dihydroxyvitamin D3, and saponins. Yang et al. (2009) assessed IM injections of sIPV adjuvanted with CpG, alum, or composite adjuvant (CpG plus alum) in mice.^90^ Significant enhancements of both humoral and cell mediated immune responses were demonstrated. In terms of dose sparing, CpG alone decreased IPV (type 2 and 3) dose by 4-fold while composite adjuvant (CpG plus alum) was more effective and led to dose reduction of IPV types 1, 2, and 3 by 4-fold, 16-fold, and 16-fold, respectively. Ghendon et al. (2011) examined IM injections of chitosan adjuvant with IMOVAX_®_ sIPV in mice.^91^ Results demonstrated significantly higher immunogenicity for adjuvanted sIPV (types 1, 2 and 3) as compared to unadjuvanted sIPV control as measured using neutralizing antibody titers in mice. High neutralizing antibody titers were obtained even with lower antigen doses and with fewer immunizations.^91^ Co-administration of 1,25-Dihydroxyvitamin D3 (DHVD3) fractionated from coconut oil, with sIPV showed significantly enhanced systemic and mucosal immunity in mice.^92^ Finally, Costa et al. (2014) studied the effects of co-administration of sIPV along with aqueous extract (AE) and saponin fraction QB-90 obtained from *Quillaja brasiliensis* in comparison to IPV adjuvanted with Quil-A adjuvant.^93^ Significant enhancements in serum concentrations of IgG, IgG1, and IgG2a were observed as compared to unadjuvanted IPV, with similar levels of enhancements obtained for IPV adjuvanted with either QB-90 or Quil-A.^93^

### IPV formulated with novel vaccine delivery systems and different routes of administration

Another vaccine formulation approach for potential cost reduction, as well as enhanced immune responses, for IPV vaccines is changing the delivery method and/or route of administration.^94^ Although IM injections are commonly used for IPV vaccinations due to their ease and repeatability, this approach requires multiple injections over time into muscle tissue relatively devoid of immune cells. Novel delivery systems that mimic multiple injections are thus of interest along with non-parenteral routes of administration. For example, mucosal delivery of IPV via sublingual or intranasal administration has been evaluated. Finally, and perhaps most promising, dermal and epidermal tissues comprise the outermost layers of the body, which are rich in immune cells. Intradermal (ID) injections of IPV could potentially elicit an immune response similar to (or better than) IM injections, but at a fraction of the vaccine dose.

As an example of formulating IPV as a single-injection vaccine to improve convenience and compliance, Tzeng et al. (2016, 2018) utilized the most-well studied and biocompatible material, poly D,L-lactic-co-glycolic acid (PLGA) based delivery system for encapsulation and controlled release of IPV *in vivo* over several weeks.^95,96^ To improve IPV stability within the PLGA polymer, IPV was co-encapsulated with basic excipients such as magnesium hydroxide and arginine to resist local, acidic pH changes caused by *in vivo* hydrolytic degradation of PLGA. Further, the addition of different amounts of a pH sensitive cationic polymer (Eudragit E PO) was useful for fine-tuning the *in vivo* pulsatile release of IPV from the PLGA polymer in two separate bursts mimicking two injections of IPV spaced a month apart. IM immunization of rats with PLGA encapsulated IPV formulation demonstrated enhanced and durable humoral immune responses in comparison to a single dose of IPV and was non-inferior to two doses of IPV injections 1 month apart.

For non-parenteral administration of IPV vaccines, Kraan et al. (2017) demonstrated the benefits of intranasal (IN) and sublingual delivery of sIPV adjuvanted with Cholera toxin vs. IM injection in mice.^97^ Antigen delivery via both mucosal delivery routes led to production of systemic polio-specific serum antibodies and neutralizing antibodies. IN delivery of sIPV adjuvanted with cholera toxin, however, significantly enhanced the neutralization titers vs. sIPV3 compared to IM delivery. Additionally, in contrast to IM administration, mucosal delivery of sIPV in the mice led to significant polio specific IgA titers at different mucosal sites including saliva, facial extracts, intestine, and IgA-producing B-cells in the spleen.^97^

### IPV vaccines and ID delivery

Historically, ID administration of vaccines has been performed by the Mantoux technique using bifurcated needles and/or multi-puncture approaches (e.g., smallpox vaccines were administered by this technique). The Mantoux technique involves dipping the needle, which comes to two solid points at the end, into the vaccine vial, followed by repeatedly pricking the skin in a small area. Multi-puncture systems use many tiny needles to puncture through the skin tissue and then apply the vaccine product to the area. This allows the vaccine to bypass the stratum corneum, a tightly joined layer of dead skin cells that forms a diffusive barrier with the environment.^98^ The layer below the stratum corneum, called the viable epidermis, allows molecules to readily diffuse through the tissue and contains Langerhans cells, which are involved in immunity and tolerance. The adjacent dermis layer also contains immune cells such as dendritic cells. These ID delivery techniques require special training of the vaccinator to reproducibly deliver the vaccine to the skin. More recently, an ID injection system for a flu vaccine using an adapter with a standard syringe has been developed and commercialized.^99^

The Salk IPV vaccine was initially developed with the intention of ID delivery; however, Denmark was the only country in which it was historically used.^100^ The Mantoux technique has been more recently used to deliver a fractional dose (one-fifth the original) that induced seroconversion in infants. The study, using the Mantoux technique in Filipino infants, compared ID and IM injections for IPV, and found that the fractional dose of IPV delivered intradermally was non-inferior to a full IM dose.^101^ A Cuban trial using a multi-puncture system to deliver an one-fifth dose of IPV produced an inferior response when compared to a full IM dose.^98^ A 2017 study found that two one-fifth fractional doses of IPV elicit a better immune response than a single IM dose.^102^ A recent 2021 dose sparing study in infants showed two fractional intradermal doses of IPV (one-fifth IPV dose) were non-inferior to two full intramuscular doses of IPV in providing adequate immunity against poliovirus type 1 and type 2, however, three intradermal doses of fractional IPV were required to provide broad immunity.^103^

Another delivery device system for ID administration of vaccines is needle-free jet injectors, which use high pressure to force the liquid through the skin (rather than a puncture using a needle).^104,105^ These devices are particularly advantageous for large-scale immunization drives in resource constrained areas.^106^ Needle-free injectors have several advantages as they eliminate needle-stick injuries, environmental burden from disposal of sharp waste generated from use of syringes and needles, logistic problems associated with needles, and could potentially be used by the patient for self-administration.^106^ Although early IM jet injections of IPV were shown to induce a similar response as IM delivery, using the same nozzle for different patients could potentially spread blood-borne disease. For example, they have been shown to transmit notable blood volumes (over 10 pl) which are sufficient to transfer diseases like hepatitis B.^106,107^ More recently, a single-use nozzle needleless injector device, the Biojector 2000, has been employed to circumvent this issue, and has been clinically studied with IPV to compare ID delivery of fractional dose with IM delivery of a full dose. A Cuban study found the fractional (1/5) dose to induce inferior seroconversion rates for all three serotypes.^108^ An Omani study found equivalent seroconversion for Types 1, 2 and 3, and in all cases, the fractional dose elicited lower antibody titers.^109^ A study with a different device in India found fractional dosing inferior as well.^110^ These limitations for jet injector ID delivery of IPV could potentially be overcome by adopting a different schedule for the fractional doses (see next section), or potentially by boosting the immune response with IPV formulated with adjuvants.

### IPV vaccines and ID delivery by microneedle patches

A newer delivery device actively being developed for ID vaccine administration is the microneedle patch. Formulating vaccines onto microneedle patches for ID delivery has the potential to enable wider coverage for a broader population.^111^ These patches are based on different technologies and manufactured in different ways, but they all involve pressing a small microarray patch (MAP) with thousands of tiny projections (50–500 µm in size) into the skin, allowing the vaccine to diffuse into the surrounding tissue.^98^ This more targeted ID delivery potentially allows sufficient or stronger immune responses at lower vaccine doses, leading to dose sparing and lower vaccine manufacturing costs in a variety of vaccine delivery applications.^111–115^ Further, unlike needles, MAPs do not result in any sharp waste leading to additional cost savings. In terms of vaccine stability, although storage of a vaccine in the dried state on MAPs can improve stability (vs. liquid formulations), they are typically sensitive to degradation in the presence of moisture during manufacturing and storage. Dissolvable MAPs thus need to be stored in a dried state prior to use.^112^ For example, Kolluru et al. (2019) demonstrated enhanced thermostability of IPV on a dissolvable microneedle device in comparison to liquid IPV, a result that may be helpful for distribution in areas with limited dependence on the vaccine cold chain.^116^

Several recent studies have examined IPV vaccines formulated in various microneedle devices. For example, Edens et al. (2015) compared microneedle-formulated sIPV at a dose of approximately 47, 9, and 38 D-antigen units of types 1, 2, and 3, respectively, to IM delivery in rhesus macaques.^113^ Immunogenicity was indistinguishable for sIPV types 1 and 2, with both routes of administration showing similar neutralizing antibody titers reaching 100% seroconversion after two doses. sIPV3 was less immunogenic for both methods of delivery, and the microneedle delivery elicited a significantly weaker immune response than IM delivery. The authors suggested that the use of an incorrect antibody for quantifying the sIPV3 could have led to an artificially insufficient dose delivered by the MAP.^113^

Several formulation studies to prepare IPV containing MAPs have been reported with the goals to minimize potency losses during manufacturing (i.e., drying the vaccine on the MAP) and storage (i.e., improved thermal stability) in comparison to conventional liquid IPV vaccines. In 2015, Kraan et al. demonstrated that the best formulations for stabilizing IPV during fabrication and storage consisted of maltodextrin and sorbitol in a histidine buffer.^75^ In another report, Kraan et al. in 2015 compared the thermostability and rat immunogenicity of trivalent IPV delivered in a liquid form (through subcutaneous or IM injection) vs. IPV delivered in a lyophilized form through using a hollow-needle MAP^98^ (through ID delivery). In accelerated stability studies, the lyophilized formulation in bioneedles was significantly more stable than liquid formulations for each IPV serotype. To simulate stresses experienced outside the vaccine cold chain, samples were taken on a 3-week trip through Middle Eastern countries. IPV Type 1 antigenicity was retained in the bioneedles while completely lost in the liquid form. The IPV Types 2 and 3 each lost 20–30% of antigenicity during the trip for both dosage forms. In rat immunogenicity studies, both dosage forms elicited similar titers when delivered individually. Furthermore, comparable retention times of 3 days for IPV at the site of injection using *in vivo* imaging were noted for both delivery methods.^98^

Finally, a 2016 dose-matching study by Muller et al. examined IPV2 delivered via a MAP system (Nanopatch^TM^) compared to IM delivery by rat immunogenicity studies. Nanopatch^TM^ formulated IPV elicited significantly higher antibody titers than the same dose given IM. When comparing seroconversion rates, a low dose delivered via Nanopatch^TM^ outperformed IM at five times the dose. A single dose of 0.2DU IPV2 (representing 1/40^th^ of full dose) via Nanopatch^TM^ led to protective antibody levels in all rats, demonstrating very high dose sparing in contrast to at least 3 immunizations required to achieve equivalent levels of neutralizing antibody titers via the IM route.^117^

**Figure 6.**
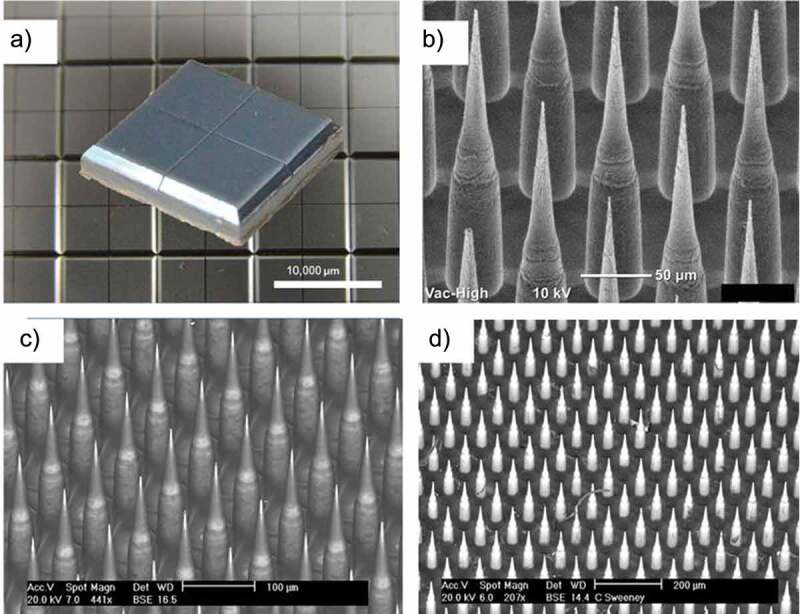
Overview of the Nanopatch^TM^ microarray patch for ID delivery of vaccines. (a) 10 mm x10 mm patch after manufacture. (b) closer look at the 250 µm needles prior to coating. (c) needles after coating with drug product where dark areas are coated and lighter areas are uncoated. (d) patch after application to rhesus monkey. Figure from Meyer et al., 2019^128^ with permission from Elsevier.

Numerous vaccine studies using Nanopatch^TM^ have been reported in preclinical studies as a promising ID delivery device.^118–128^ Figure 6a–d show images of Nanopatch^TM^ device and its needles before and after coating them with antigen and application. The Nanopatch™ comprises a 1 cm^2^ silicon chip-like patch with high-density micro-projections on the skin-facing side to target the vaccine to antigen-presenting cells in the epidermal and dermal layers of the skin.^117,129,130^ The combined effect of targeted delivery and inflammation due to cell death leads to improved immunity vs. needle-based vaccine delivery.^117,131^ In the newer version of the Nanopatch™ technology, the manufacturer Vaxxas has made significant progress toward the use of a polymer-based patch.

### Formulation development challenges with IPV vaccines and Nanopatch™

**Figure 7.**
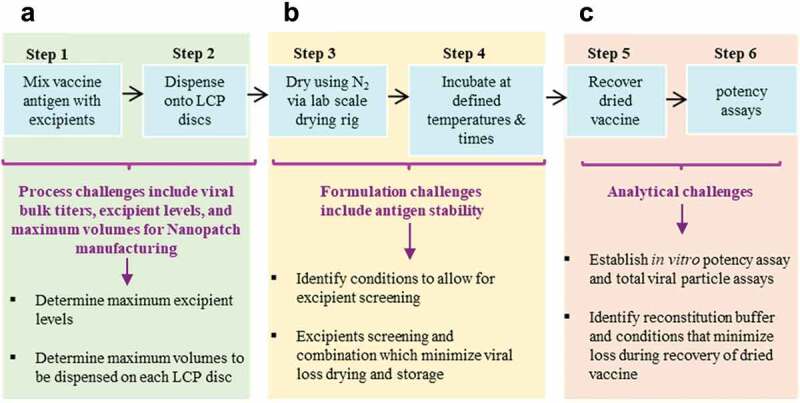
Overview of experimental challenges encountered during development of stable, dried formulations of vaccine candidates for use in the Nanopatch^TM^ delivery system. (a) manufacturing process constraints for amounts of excipients and antigens available, (b) formulation challenges to identify stabilizing additives, and (c) analytical challenges to measure recovery and stability of vaccine antigens. This figure describes a lab-based, scaled-down model of the Nanopatch^TM^ process and is adapted from open access article by Wan et al., 2021^132^.

Formulation development of IPV antigens with Nanopatch™ technology is a critical step to enable the potential advantages of microneedle delivery into a viable commercial vaccine dosage form. The three IPV antigens (Types 1, 2, 3) can undergo different stresses during Nanopatch™ manufacturing and delivery that can affect their stability, potency, and costs to manufacture. First, the maximum vaccine dose and acceptable excipient levels that can be employed have limitations, based on constraints from bulk IPV and Nanopatch™ dosage form manufacturing processes (Figure 7a).^132^ This in turn presents vaccine stabilization and analytical development challenges (Figure 7b,c), respectively, including (1) screening for stabilizing pharmaceutical excipients (and their combinations at optimal concentrations) to minimize antigen loss during drying and subsequent storage in the dried state, and (2) developing stability-indicating analytical methods and experimental conditions to monitor antigen yields and stability during formulation development.^132–136^

**Figure 8.**
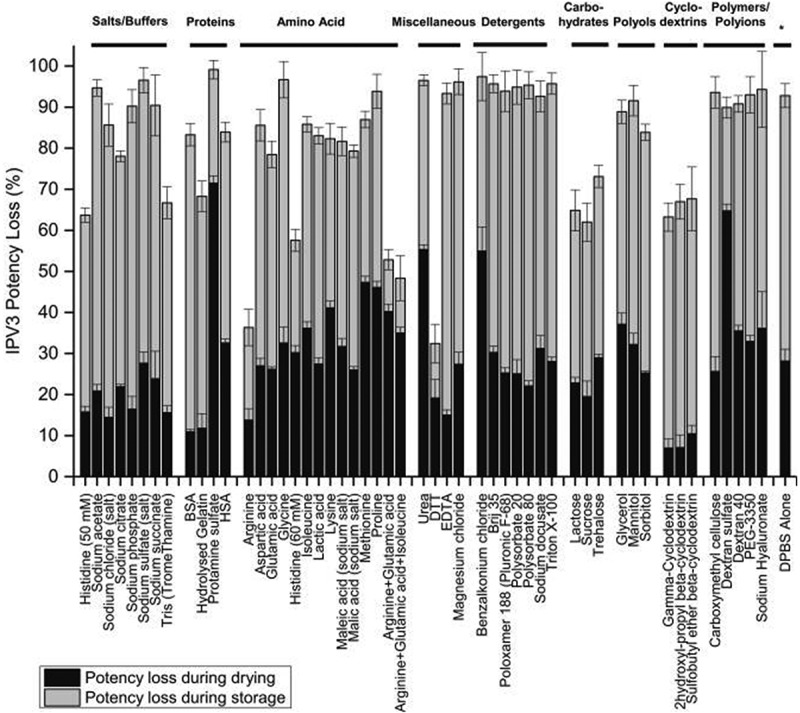
Effect of excipient category and type on *in vitro* potency losses after drying and storage of the IPV3 component of trivalent-IPV samples using scale-down model of Nanopatch^TM^ delivery system. Each condition is shown as a relative percentage D-antigen values compared to a control (liquid tIPV stock solution), and the black and gray bars denote relative losses of D-antigen values during drying and storage for 7 days at 4°C on LCP discs. Error bars represent one SD range from quadruplicate experiments. Figure from Wan et al., 2018^28^ with permission from Elsevier.

**Figure 9.**
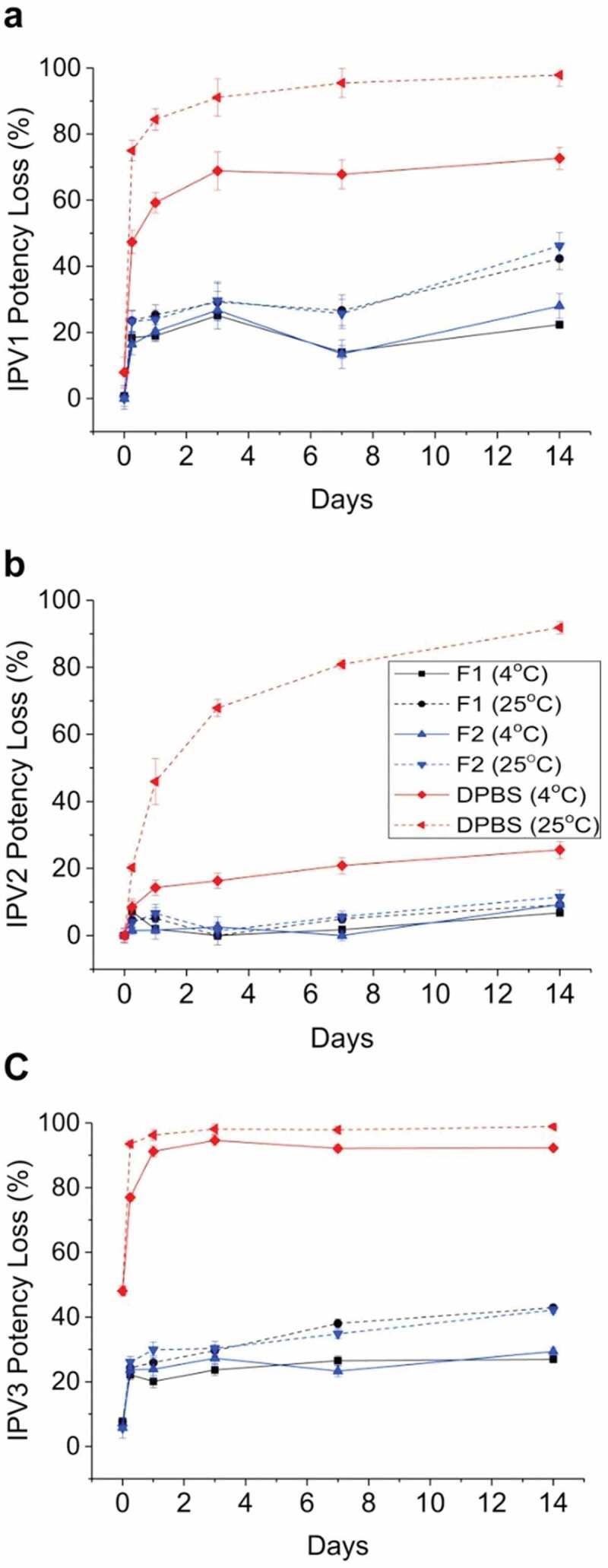
Stability profile of trivalent IPV in two candidate formulations during short-term storage in the dried state using scale-down model of Nanopatch^TM^ delivery system. Relative D-antigen values were measured for each IPV serotype (a) IPV1, (b) IPV2, (c) IPV3, and are relative to an unstressed liquid t-IPV stock solution. The T0 values display IPV serotypes losses immediately after drying. The t-IPV samples were dried onto LCP discs and stored in candidate formulations in a base buffer containing PBS and M199 with the following additives (F1, black): 4.5% SBE-beta-cyclodextrin +15-mM glutathione, (F2, blue): 2.5% gamma-cyclodextrin +15-mM glutathione), or (DPBS, red): no additional excipients. Samples were stored at either 4°C (solid lines) or 25°C (dashed lines) for up to 14 days and bars represent the one SD from triplicate experiments. Figure from Wan et al., 2018^28^ with permission from Elsevier.

The development of candidate formulations capable of stabilizing a trivalent IPV (t-IPV) vaccine during drying and storage in the Nanopatch^TM^ delivery system, as prepared using a scaled-down lab model of the MAP manufacturing process and analyzed using optimized D-antigen potency assays, was described by Wan et al. in 2018.^28^ The loss of IPV D-antigen values after drying was found to only partially be due to the drying stress, but also due to incomplete recovery in the assay. A combination of 0.1% (v/v) PS-80 and 1% (w/v) BSA in PBS buffer prevented nonspecific adsorption and maximized recovery of trivalent IPV antigens from the LCP disc in the assay. Next, excipient screening studies with ~50 pharmaceutical excipients, at various concentrations, were employed with t-IPV after drying and storage at 4°C for 7 days. The results identified stabilizing excipients including reducing agents (e.g., dithiothreitol), certain amino acids (e.g., arginine and histidine), carbohydrates (e.g., sucrose or lactose), and cyclodextrins (e.g., ɣ-cyclodextrin, 2-OH propyl β-cyclodextrin, and SBE-β-cyclodextrin) (Figure 8). Promising additives were further evaluated at various concentrations and combinations to prepare optimized formulations. Interestingly, *in vitro* potency losses in the optimized formulations occurred primarily during the initial few weeks of storage at 4°C and 25°C with a leveling off afterward. The stability profiles of two candidate optimized formulations containing trivalent IPV (vs. a DPBS buffer control) were evaluated at 4°C and 25°C for 2 weeks post-drying (Figure 9). The relative D-antigen potency losses of each of the three IPV antigens in DPBS control were very notable at 25°C with greatly improved stability observed in the two candidate formulations containing cyclodextrin and reduced glutathione.

## Conclusions and applications of IPV/sIPV formulation development “lessons-learned” to other vaccines

In this review, the challenges and opportunities for vaccine formulation development to further lower costs and increase coverage of inactivated polio vaccines were examined, especially as vaccine manufacturers continue ongoing efforts to replace the trivalent Salk IPV antigens (produced from wild-type polioviruses) with Sabin IPV antigens (produced from attenuated polioviruses employed in OPV). Sabin IPV is non-inferior to Salk IPV in terms of immunogenicity, and widespread implementation of Sabin IPV will facilitate improved biosafety and lowered costs during vaccine manufacturing. Low-cost formulation approaches include development of IPV/sIPV vaccine multidose formulations (more doses per vial), combination vaccine formats (more vaccine antigens per dose), as well as improving storage stability in the vaccine cold chain. Moreover, employing formulation strategies for IPV/sIPV dose-sparing and enhanced mucosal immunity were highlighted including adjuvants (e.g., aluminum-salt and newer adjuvants), and delivery systems (e.g., ID administration with microneedle patches). Together, new formulations of the sIPV-based polio vaccine can enable cost-reduction and expand access to LMICs to aid in the overall goal of worldwide polio eradication.

Importantly, IPV/sIPV formulation development approaches reviewed herein can be applied to other vaccines targeted for use in LMICs including those with similar antigen platforms (e.g., live-attenuated, or inactivated viruses) as well as newer ones (subunit-protein, viral vectors, or mRNA). Two promising examples where improved vaccine formulations could help to further reduce disease burden and mortality in LMICs include human papillomavirus (HPV) and rotavirus (RV) vaccines. Both vaccines are currently widely used in high-income countries, but due to their high cost and limited production capacity, their availability and coverage are suboptimal in LMICs. For HPV vaccines, although highly successful in high-income countries in reducing the incidence of cervical cancer and other HPV-related diseases, HPV vaccines are not as widely available in LMICs.^137,138^ For example, in the year 2020, ~604,000 cervical cancer cases, and ~342,000 deaths, were reported globally, with ~90% occurring in LMICs.^139^ For RV vaccines, tens of millions of infants remain unvaccinated in LMICs due to limited manufacturing capacity, high vaccine and implementation costs, lower efficacy in LMICs (>80% vs. >50%), and potential safety concerns such as intussusception.^140^ Rotavirus disease remains the leading cause of acute gastroenteritis in children <5 years of age, leading to estimated 215,000 worldwide deaths annually, mainly in the LMIC.^141^

To implement more affordable HPV vaccines for use in LMICS, new and/or improved manufacturing and formulation platforms are needed,^58^ and one such approach is developing multi-dose formulations to lower the costs associated with manufacturing, packaging, storage, and distribution.^142^ While IPV vaccines contain inactivated viral particles made from growing the virus in bioreactors, HPV vaccines contain virus-like particle (VLP) antigens each consisting of recombinantly expressed L1 virus surface proteins that self-assemble into VLPs.^143^ Interestingly, both IPV and HPV antigens are incompatible with the commonly used vaccine preservative thimerosal (TH). Trivalent IPV vaccines from various manufacturers are each compatible with an alternative vaccine preservative (2-phenoxyethanol). In contrast, different HPV vaccines (consisting of HPV VLP antigens from unique expression systems, purifications, and formulations) each display different stability profiles in the presence of different preservatives, a situation that has led to the requirement of customized approaches for multidose HPV vaccine formulation development.^54,144^ Compared to standalone trivalent IPV vaccines, formulation development of multidose HPV vaccines is even more challenging since they not only contain aluminum adjuvants but also between two and nine different HPV VLP antigens with varying sensitivities to preservatives. Currently, no multidose HPV vaccines are commercially available.

For RV vaccines, four live-attenuated, orally administered RV vaccines are now commercially available including the RotaTeq® and Rotarix® vaccines developed in the 2000s and two newer RV vaccines that have recently received WHO prequalification status (Rotavac® and ROTASIIL®). Nonetheless, improved, next-generation RV vaccines are being developed for use in LMICs to lower costs, expand access, and hopefully improve efficacy, including two vaccine design approaches: (1) new live attenuated viruses for oral delivery, and (2) new subunit protein-based antigens for parenteral administration.^145^ For the first approach, two formulation goals to lower costs and improve accessibility in LMICs include (1) good storage stability in the vaccine cold chain of a liquid, refrigerator stable formulation, and (2) oral administration without the need for a separate step of preneutralization of gastric acid. Such RV vaccine formulations would improve upon the OPV vaccine distribution cold-chain that requires the liquid formulation to be stored frozen. As an example, process and formulation challenges encountered during the development of RV3-BB, a live-attenuated, oral RV vaccine candidate in late-stage clinical trials targeted for use in LMICs,^136,146^ have been recently reported.^133,134,136,147^

As described in this review, the transition from orally administered, live OPV to parenterally administered, inactivated IPV/sIPV antigens allows for the inclusion of polio vaccines into routine pediatric combination vaccines employed in LMICs containing DTwP, HepB, and Hib. Inclusion of IPV antigens into combination vaccines lowers costs and increases vaccine coverage and could play a crucial role in providing polio immunity worldwide. Based on these considerations, a parenterally administered, non-replicating rotavirus vaccine (NRRV) candidate is being developed by PATH consisting of a trivalent mixture of three recombinant protein antigens formulated with aluminum adjuvant.^145,148^ NRRV is currently in late-stage clinical trials and has been shown to be effective in early clinical trials with the potential to overcome the high costs and limited production capacity of currently available rotavirus vaccines in LMICs.^145,148^ Recent economic studies have highlighted the substantial cost saving potential of adding the NRRV antigens to routine pediatric combination vaccines containing DTP, HepB, and Hib.^149^ Similar to IPV antigens, however, the three NRRV antigens are destabilized by thimerosal, limiting their potential use in multidose, wP-containing pediatric combination vaccines.^66,67^ Furthermore, guinea pig immunogenicity studies demonstrate that Alhydrogel®-adsorbed NRRV antigens provide the highest neutralization titers, suggesting that the adjuvant-adsorbed state of NRRV antigens will be an important formulation goal for retaining immunogenicity in pediatric combination vaccines.^150^ The compatibility and stability evaluations of trivalent NRRV antigens in the presence of various antigens found in pediatric combination vaccines (e.g., DTwP, Hib, HepB, and IPV) are currently ongoing in our laboratories (manuscript in preparation).

This review also highlights the critical role of stability-indicating *in vitro* potency methods (D-antigen ELISA), which correlate well with *in vivo* potency assays (rat immunogenicity), to enable successful IPV vaccine formulation development. In the case of IPV, the D-antigen assay was shown to be more sensitive to structural changes in the IPV antigens compared to animal immunogenicity results, demonstrating its utility as an early-indicator of vaccine instability. Similar *in vitro* vs. *in vivo* potency assay correlations have been established with HPV vaccines and were recently demonstrated for NRRV antigens.^135^ Moreover, similar analytical challenges described herein with IPV antigens during formulation development have been encountered with other vaccines. For example, the numerous analytical challenges identified and successfully addressed when formulating IPV vaccines into microneedle array patches (MAPs) for ID administration (as described above), have been applied to successfully stabilize live measles and rubella vaccines being reformulated in MAPs.^132^

This review highlights the crucial role that vaccine analytical and formulation development plays in producing low-cost, widely available, stable vaccines, which in turn facilitates worldwide immunization efforts to mitigate vaccine preventable infectious diseases. As a final example, many of the “lessons-learned” described in this review during the formulation of development of low-cost IPV dosage forms can also be applied to the development new COVID-19 vaccine candidates targeted for use in LMICs.^151^ Next-generation COVID-19 vaccines for use in LMICs are needed to address current limitations in terms of immune responses (e.g., durability and breadth) and manufacturing (e.g., lower costs, expanded production capacity, and improved stability) compared to currently available mRNA-based COVID-19 vaccines. As an example, leveraging the experience with IPV vaccine formulation development described herein, we recently reported the critical importance of developing sensitive, stability-indicating *in vitro* potency assays to better understand the inter-relationships of antigen-adjuvant interactions, storage stability, and *in vivo* animal immunogenicity profiles for a low-cost subunit COVID-19 vaccine candidate (SARS-COV-2 receptor-binding domain (RBD) antigen formulated with different adjuvants).^152^

## Abbreviations

AF4-MALS: asymmetric flow field-flow fractionation with multi-angle light scattering
BSA: bovine serum albumin
IPV: conventional formalin-inactivated polio virus
COVID-19: coronavirus disease 2019
cVDPV: circulating vaccine-derived polio virus
DCVM: developing countries vaccine manufactures
DLS: dynamic light scattering
dmLT: double mutant heat-labile enterotoxin
DPBS: Dulbecco’s phosphate-buffered saline
DSC: differential scanning calorimetry
DTP: diphtheria tetanus pertussis vaccine
DTT: dithiothreitol
DU: D-antigen unit
ELISA: enzyme-linked immunoassay
Hep B: hepatitis B
Hib: *Haemophilus influenzae*type b
ID: intradermal
IM: intramuscular
IPV: inactivated polio virus
LMIC: low- and middle- income countries
MDVP: multi-dose vial policy
N/S: needle and syringe
NRRV: non-replicating rotavirus vaccines
OPV: oral polio vaccine
2-PE: 2-phenoxyethanol
PLGA: poly D,L-lactic-co-glycolic acid
PV: polio Virus
RNA: ribonucleic acid
SDU: Sabin D-antigen unit
sIPV: Sabin inactivated polio virus
SPR: surface plasmon resonance
SV-AUC: sedimentation velocity analytical ultracentrifugation
TEM: transmission electron microscopy
VAPP: vaccine associated paralytic poliomyelitis
VP: viral proteins
VVM: vaccine vial monitor
WHO: World Health Organization
wP: whole cell pertussis
